# The Ultimate Buzzkill: When Dengue Hits Hard

**DOI:** 10.7759/cureus.80761

**Published:** 2025-03-18

**Authors:** Neema Francis, Preethi Asapu, Mashhood U Qazi, Thiagarajan Jaiganesh

**Affiliations:** 1 Emergency Medicine, Tawam Hospital, Al Ain, ARE; 2 Emergency Medicine, Sheikh Tahnoon Bin Mohammed Medical City, Al Ain, ARE

**Keywords:** cardiac arrest, dengue, flu, hemorrhagic fever, mosquito

## Abstract

Dengue cases have been rising globally in recent years. In the United Arab Emirates (UAE) specifically, weather conditions have created an ideal breeding ground for the Aedes aegypti mosquito, the primary vector of the disease. We report the case of a 62-year-old South Asian male residing in the UAE who initially experienced vague flu-like symptoms. His condition progressively worsened, ultimately resulting in an unexpected out-of-hospital cardiac arrest and, tragically, death.

## Introduction

Infection caused by dengue is rising alarmingly, with over 14 million dengue cases and around 10,000 deaths reported in 2024 [1]. Dengue, a mosquito-borne viral infection, is a disease that is spread by the Aedes aegypti mosquito [2]. It can present with a spectrum of symptoms ranging from completely asymptomatic patients to severe manifestations such as dengue hemorrhagic fever and dengue shock syndrome (DSS). Although cardiac complications are generally rare, they are becoming increasingly recognized in severe dengue cases [3]. In 2024, 7.6 million dengue cases were reported to the World Health Organization (WHO), which included 3.4 million confirmed cases, over 16,000 severe cases, and more than 3,000 mortalities [4]. We describe a rare and critical presentation of dengue-related cardiac arrest in a male patient, aiming to highlight the importance of early recognition and aggressive management of severe complications in dengue fever.

## Case presentation

An unconscious 62-year-old male patient was brought to the Tawam Hospital, Emergency Department (ED) by the Emergency Medical Service (EMS). On arrival, he was noted to be pulseless. Collateral history was obtained from the attendants, who stated that he had been feeling unwell for the past three days and had been seen in a nearby clinic earlier in the day with flu-like symptoms. He had been discharged with symptomatic management. After discharge, he had complained of feeling severely unwell and hence been advised to attend the ED. They further reported that the patient had not traveled anywhere recently; however, two people from his shared residence had similar symptoms. His acquaintances reported that he had become unconscious in the passenger seat of the vehicle when they had been 10 minutes away from the hospital; however, no bystander cardiopulmonary resuscitation (CPR) had been initiated.

**Figure 1:**
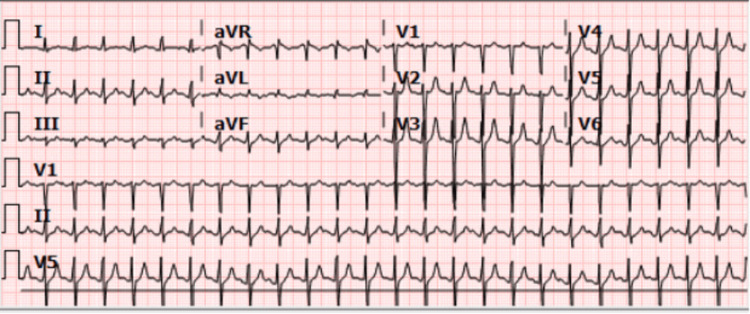
ECG showing sinus tachycardia ECG: electrocardiogram

Upon the patient's arrival at the ED, CPR was initiated as per the advanced cardiovascular life support (ACLS) protocol, and he was rushed to the resuscitation area. The initial rhythm on the cardiac monitor revealed asystole, and his pupils were fixed and dilated. The patient was intubated, and a venous blood gas (VBG) revealed severe metabolic acidosis, with a lactate of 22 mmol/L. After four cycles of CPR, return of spontaneous circulation (ROSC) was achieved, and an ECG (Figure 1) showed sinus tachycardia. Post-ROSC management was initiated and an orogastric tube and an arterial line were inserted. 

**Figure 2:**
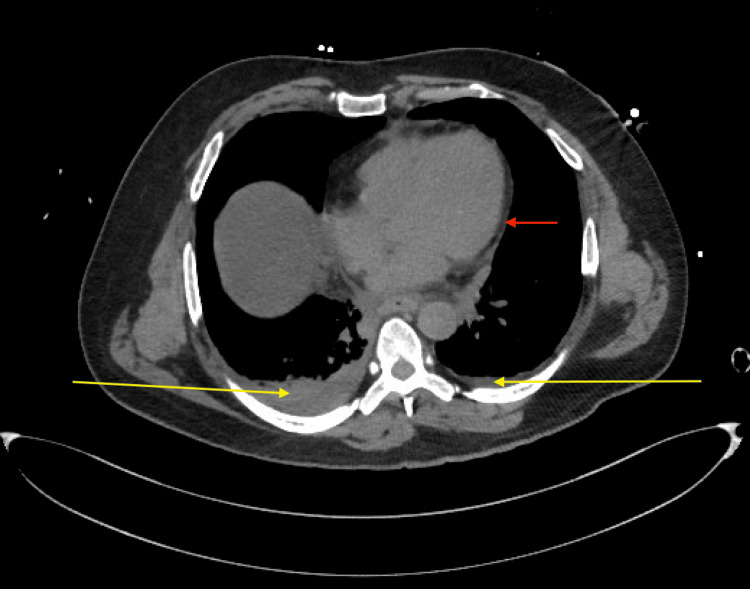
CT chest axial view The scan revealed bilateral mild pleural effusion, more pronounced on the right side (yellow arrows). A mild pericardial effusion was observed (the red arrow) CT: computed tomography

Despite intravenous fluid resuscitation, the patient was severely hypotensive and required vasopressor support with norepinephrine infusion. A chest radiograph was unremarkable. His blood test results revealed a critically low platelet count, which prompted the team to send out a dengue screen. Additionally, a pan CT revealed mild, bilateral pleural effusions as well as a mild pericardial effusion (Figure 2). He was then admitted to the ICU for post-cardiac arrest care, exhibiting severe metabolic acidosis, shock, and anoxic brain injury. Further imaging and bedside assessments indicated possible pancreatitis and non-significant pleural and peritoneal fluid collections. His prognosis remained guarded with a high likelihood of early mortality. He remained hypotensive in the ICU and subsequently experienced another cardiac arrest. ROSC was achieved after three cycles of CPR, and post-ROSC management was initiated. Although high doses of norepinephrine and vasopressin were administered as well, he suffered another cardiac arrest, and unfortunately, ROSC was not achieved on this occasion. A few days later, his dengue panel returned positive.

## Discussion

Dengue has become a significant public health challenge due to a marked increase in reported cases over the past two decades. In 2024, 7.6 million dengue cases were reported to WHO, which included 3.4 million confirmed cases, over 16,000 severe cases, and more than 3,000 mortalities [4]. Dengue virus (DENV) is an enveloped virus with a single positive-stranded RNA genome that belongs to the genus Flavivirus of the family Flaviviridae and consists of four serotypes: DENV-1, DENV-2, DENV-3, and DENV-4 [5,6]. The disease manifests in three stages. The febrile phase consists of flu-like symptoms, followed by the critical phase, which could have varying degrees of plasma leakage. This leads to intravascular volume depletion and might progress to life-threatening DSS and, subsequently, the convalescent phase, where plasma leakage ceases and reabsorption commences [7,8]. 

DSS is relatively more common in patients who have been infected by the virus at a previous encounter, and this is due to an immunopathological mechanism that is triggered by sequential infections with different viral serotypes [9]. DENV-2 and DENV-3 serotypes can directly or indirectly affect cardiac tissue through the activation of an immunological cascade. Both processes occur in conjunction, leading to severe complications from the disease [10]. Numerous complications such as arrhythmias, heart blocks, endocarditis, and myocarditis can arise from cardiac involvement but these are seldom associated with life-threatening consequences [10].

Our patient’s ECG showed sinus tachycardia, which is the most common arrhythmia recognized in these cases. Pathophysiologically, the disease is usually mild and self-limited but can escalate to severe heart failure with global hypokinesia and cardiac dilation. The mechanism begins with DENV's attack on macrophages, which in turn triggers T-cell activation [11,12,13]. Activated T cells then release inflammatory mediators and activate complement system pathways (C3a and C5a), causing inflammation and necrosis of endothelial cells, which increases capillary permeability and leads to vascular leakage. This leakage into myocyte interstitial spaces results in myocardial interstitial edema, impairing myocardial contraction and reducing overall cardiac function. Additionally, intravascular blood volume decreases, impairing coronary circulation. Cardiac electrical conduction is affected by cytokine release, which suppresses cardiac contractility and can lead to conduction blocks and arrhythmias [14,15,16].

The main management goals rely on early detection of myocardial damage to prevent catastrophic complications such as multiorgan failure and death. Diagnostic methods like cardiovascular magnetic resonance (CMR) and endomyocardial biopsy can be useful in diagnosing myocarditis, though their effectiveness may be limited. When myocarditis is suspected, elevated serological markers of cardiac injury, along with changes in ECG and echocardiograms, can help in its identification, as observed in our patient [17]. Our patient's blood test results showed elevated troponin levels and the CT scan illustrated mild pericardial effusion and pleural effusions. The cornerstone of management predominantly depends on early recognition and vigilant monitoring of the degree of hemodynamic instability and involves the timely identification of cardiac abnormalities followed by appropriate supportive care [18]. Fluid resuscitation is crucial for managing DSS, as it aims to restore intravascular volume, maintain organ perfusion, and counteract plasma leakage. The challenge lies in balancing adequate fluid replenishment while avoiding fluid overload [19].

The management of DSS is rapidly advancing, driven by research and the development of innovative treatments. Antiviral medications currently ongoing clinical trials are being evaluated for their effectiveness and safety, making targeted antiviral therapy increasingly feasible. The introduction of dengue vaccines, designed to provide immunity against all four dengue serotypes, represents a paradigm shift in DSS management [20].

## Conclusions

Most cardiac complications arising from dengue fever are self-limiting; however, in rare cases, they can progress to severe outcomes such as DSS and cardiac arrest, as observed in this patient. The presence of severe metabolic acidosis, hypotension, and pericardial effusion in this case highlights the potential for dengue to cause multi-organ dysfunction, including cardiovascular collapse. Early recognition and aggressive management of cardiac involvement in dengue are crucial, as timely intervention may improve patient outcomes. Given the increasing incidence of severe dengue cases globally, further research is needed to establish standardized protocols for diagnosing and managing dengue-related cardiac complications. This report underscores the need for heightened clinical awareness of cardiovascular involvement in dengue fever, particularly in critically ill patients.
